# Unveiling prognostic factors in neonatal modified Blalock-Taussig shunt procedures: a comprehensive single-center analysis

**DOI:** 10.1590/1806-9282.20252127

**Published:** 2026-06-23

**Authors:** Hasan Akduman, Dilek Dilli, Başak Kaya, İlker Mercan, Özkan Kaya, Ayşegül Zenciroğlu

**Affiliations:** 1 University of Health Sciences of Türkiye, Ankara Etlik City Hospital, Department of Neonatology – Ankara, Türkiye.; 2 University of Health Sciences of Türkiye, Ankara Etlik City Hospital, Department of Pediatric Cardiovascular Surgery – Ankara, Türkiye.; 3 University of Health Sciences of Türkiye, Ankara Etlik City Hospital, Department of Pediatric Cardiology – Ankara, Türkiye.

**Keywords:** Heart defects, congenital, Infant, newborn, Cardiac surgical procedures, Risk factors, Mortality

## Abstract

**OBJECTIVE:**

The aim of this study was to describe early outcomes after neonatal modified Blalock-Taussig shunt and identify perioperative predictors of mortality in a tertiary congenital cardiac center.

**METHODS:**

In this retrospective cohort, 129 neonates with critical congenital heart disease who underwent modified Blalock-Taussig shunt were analyzed. Demographic, clinical, and perioperative variables were collected, and multivariate logistic regression was used to identify independent predictors of neonatal mortality.

**RESULTS:**

Overall neonatal mortality was 21.7%. Shunt failure occurred in 18.9% (24/127) of neonates transferred to the neonatal intensive care unit and was strongly associated with mortality (66.7 vs. 11.6%; p=0.001). Independent risk factors for neonatal mortality included lower gestational age (OR 0.82, 95%CI 0.72–0.94; p=0.004), preoperative inotropic support (OR 3.46; p=0.006), preoperative mechanical ventilation (OR 4.28; p=0.001), shunt failure (OR 5.12; p=0.001), and postoperative metabolic acidosis (OR 6.78; p=0.001).

**CONCLUSION:**

Neonates undergoing modified Blalock-Taussig shunt remain at high risk for postoperative complications and death. Preoperative hemodynamic instability, shunt failure, and postoperative metabolic acidosis are strong predictors of poor outcome. These findings support vigilant perioperative monitoring and individualized management strategies in neonatal modified Blalock-Taussig shunt candidates.

## INTRODUCTION

Congenital heart disease (CHD) is a major cause of neonatal morbidity and mortality, with a global birth prevalence of approximately 0.94% of live births^1^. Critical CHD (CCHD) accounts for roughly one quarter of CHD cases and usually requires early surgical or catheter-based intervention. Despite advances in surgery and perioperative care, mortality in this group remains considerable^2^.

The modified Blalock-Taussig shunt (MBTS) is a widely used palliative procedure that has largely replaced the classical shunt because of its relative safety and its favorable effects on pulmonary artery growth. However, MBTS is associated with important complications, including chylothorax, shunt thrombosis, and distortion of the pulmonary arteries^3^. It is most often employed in neonates with duct-dependent cyanotic lesions, such as pulmonary atresia, severe pulmonary stenosis, tetralogy of Fallot, and tricuspid atresia, in whom primary repair is frequently not feasible in the neonatal period. In these settings, MBTS serves as a bridge to staged procedures such as the Glenn and Fontan operations^4^.

Several clinical and perioperative variables have been proposed as prognostic factors after MBTS, including shunt-to-weight ratio, gestational age, preoperative mechanical ventilation, shunt thrombosis, and renal dysfunction^5^. The present study aims to describe short- and intermediate-term outcomes of neonates undergoing MBTS in a high-volume congenital cardiac center and to identify key predictors of mortality and morbidity, with the goal of improving perioperative risk stratification and guiding future management strategies.

## METHODS

### Study setting and patient selection

This retrospective cohort study was conducted in the Level III neonatal intensive care unit (NICU) of a tertiary referral center for CHD in Ankara, Türkiye. All neonates with critical CHD who underwent a MBTS between January 2011 and December 2021 were included. Neonates with major non-cardiac congenital anomalies that precluded surgery and those who died before MBTS were excluded.

### Data collection

Data were retrieved from electronic medical records and included demographic characteristics (gestational age, birth weight, sex, mode of delivery), primary cardiac diagnosis, genetic findings, timing of surgery, preoperative hemodynamic status (need for mechanical ventilation and inotropic support), perioperative events, and clinical outcomes. Laboratory variables comprised the highest hematocrit, lowest serum albumin, and highest serum creatinine levels within 48 h before and after surgery. Metabolic acidosis was recorded if present within the first 24 postoperative hours.

### Surgical procedure

MBTS was performed via left or right lateral thoracotomy. A polytetrafluoroethylene (PTFE) or homograft conduit was anastomosed between the subclavian and pulmonary arteries. When a homograft conduit was used, the conduit diameter was selected using the same weight-based criteria applied to PTFE grafts: 3.0 mm for neonates <2.5 kg, 3.5 mm for 2.5–3.5 kg, and 4.0 mm for >3.5–4.0 kg^3,6^. Homograft use was limited to selected cases based on intraoperative considerations and surgeon discretion/availability, and the conduit length was tailored to achieve a tension-free anastomosis with optimal alignment of the subclavian and pulmonary arteries. Operative times varied between 90 and 150 min depending on anatomical complexity^7^.

Intravenous unfractionated heparin (UFH; 10–15 IU/kg/h) was initiated within 6 h postoperatively. In selected cases, low-molecular-weight heparin (LMWH; 1.5 mg/kg every 12 h) was used. Following initiation of enteral nutrition, patients were transitioned to oral acetylsalicylic acid (3–5 mg/kg/day), particularly in cases with graft diameters ≤4 mm^8,9^.

### Neonatal intensive care unit follow-up and outcome measures

Hemodynamically stable infants were transferred to the NICU within 2 h of surgery; unstable patients remained in the cardiac surgery ICU until stabilization. Mechanical ventilation used pressure-controlled modes, targeting preductal oxygen saturation of 75–85% and normocapnia, while avoiding hyperoxia and alkalosis. Extubation was considered when hemodynamics and shunt function were stable and spontaneous breathing was adequate. Shunt failure was defined as complete occlusion or critical narrowing, indicated by sudden desaturation and loss of shunt murmur, confirmed by transthoracic echocardiography with markedly reduced or absent flow. Postoperative morbidities included pulmonary complications (pneumonia, hemorrhage, overcirculation), neurological events (intracranial hemorrhage, seizures, periventricular leukomalacia, hypoxic–ischemic encephalopathy), significant bleeding requiring intervention, infections (culture-positive sepsis or surgical site infection), and other complications such as acute kidney injury, multiple organ dysfunction syndrome, necrotizing enterocolitis (Stage≥II by modified Bell’s criteria), and diaphragmatic paralysis confirmed by ultrasonography or fluoroscopy.

Early neonatal mortality was defined as death within the first 30 days of life or before NICU discharge. Late mortality refers to death after NICU discharge but before second-stage surgical palliation.

### Ethical approval

The study was approved by the Institutional Ethics Committee of the University of Health Sciences, Ankara Dr. Sami Ulus Maternity and Children’s Health and Diseases Training and Research Hospital (Approval No: 2021/02-088). Owing to the retrospective design, the requirement for informed consent was waived. The study complied with the Declaration of Helsinki and national ethical regulations.

### Statistical analysis

Data analysis was conducted using IBM SPSS Statistics for Windows (Version 24.0, IBM Corp., Armonk, NY, USA). The Kolmogorov-Smirnov test assessed the normality of continuous variables. Categorical variables were expressed as frequencies and percentages; continuous variables were presented as mean ± tandard deviation or median (range), based on distribution.

Chi-square or Fisher’s exact test was used to compare categorical variables. Independent samples t-test or Mann-Whitney U test was applied for continuous variables, as appropriate. Multivariate logistic regression was performed to identify independent predictors of neonatal mortality. Variables included in the model were selected based on clinical significance and univariate analysis (p<0.1), and included gestational age, birth weight, preoperative hematocrit, albumin, inotropic and ventilatory support, postoperative metabolic acidosis, and shunt failure. Odds ratios (OR) with 95%CI were calculated, with statistical significance defined as p<0.05.

## RESULTS

During the study period, 930 neonates with CHD were admitted to the NICU. After multidisciplinary evaluation, 137 were considered to require MBTS. In total, eight neonates (four with severe hemodynamic instability, two with sepsis, and two with multiple major congenital anomalies) were excluded, leaving 129 neonates who underwent MBTS and constituted the study cohort. Of these, 61 (47.2%) had single-ventricle physiology and 68 (52.7%) had biventricular physiology. Two neonates died intraoperatively; therefore, postoperative outcomes and shunt failure were assessed in the remaining 127 neonates transferred to the NICU. Cardiac diagnoses are summarized in Table 1.

**Table 1:** Subtypes of congenital heart diseases in infants undergoing modified Blalock-Taussig shunt.

Subtype of congenital heart disease	n=129 (%)
PA/IVS	83 (64.3)
Tricuspid atresia	15 (11.6)
DORV+PS	11 (8.5)
TOF	10 (7.8)
Complete AVSD+PS	5 (3.9)
TGA	3 (2.3)
DILV	2 (1.6)

AVSD: atrioventricular septal defect; CHD: congenital heart disease; DILV: double inlet left ventricle; DORV: double outlet right ventricle; IVS: intact ventricular septum; MBTS: modified Blalock-Taussig shunt; NICU: neonatal intensive care unit; PA/IVS: pulmonary atresia with intact ventricular septum; PS: pulmonary stenosis; SD: standard deviation; TGA: transposition of great arteria; TOF: tetralogy of Fallot.

The mean gestational age was 37.9±1.98 weeks, and the mean birth weight was 2,909±532 g. The most common presenting features were cyanosis, cardiac murmur, oxygen desaturation, and respiratory distress. Prenatal diagnosis was available in 21.7% of patients, and one infant (0.7%) was detected by routine pulse oximetry screening. Demographic and baseline clinical characteristics are shown in Table 2.

**Table 2:** Demographic and clinical characteristics of all study patients.

Variables	n=129
Gestational age (weeks), mean±SD	37.9±1.98
Birth weight (grams), mean±SD	2,909±532
Prematurity (<37 weeks), n (%)	11 (8.5)
Low birth weight (<2,500 g), n (%)	7 (5.4)
Gender, n (%)—female	67 (52)
Mode of delivery, n (%)—(C/S)	66 (51.2)
Prenatal diagnosis, n (%)	28 (21.7)
Age on admission (days), median (min–max)	2 (1–35)
Age at diagnosis (days), median (min–max)	1 (1–35)
Time to MBTS surgery (days), median (min–max)	16 (1–360)
Shunt failure, n (%)	24 (18.9)
Length of NICU stay, (days), median (min–max)	33 (7–211)
Neonatal mortality, n (%)	28 (21.7)

Shunt failure was assessed among neonates transferred to the NICU (n=127); percentages for shunt failure are calculated using n=127 as the denominator. C/S: cesarean section; MBTS: modified Blalock-Taussig shunt; NICU: neonatal intensive care unit; SD: standard deviation.

Chromosomal anomalies were identified in nine patients (6.9%), most frequently trisomy 21 (n=6). Other syndromes included trisomy 18, DiGeorge syndrome, and a 5q35 deletion. One infant born to a mother with phenylketonuria had complex CHD with double outlet right ventricle, pulmonary atresia, and atrioventricular septal defect.

Before MBTS, 99 infants underwent diagnostic cardiac catheterization; 29 (22.5%) received additional interventions, such as balloon atrial septostomy, valvuloplasty, wire perforation of an intact ventricular septum, or ductal stenting. Prostaglandin infusion was administered in 110 infants (84.9%), inotropic support was required in 34%, and mechanical ventilation in 37.7%. Preoperative sepsis, arrhythmia, and acute kidney injury requiring dialysis were observed in 13.2, 5.7, and 1.9% of patients, respectively.

## Postoperative outcomes and long-term follow-up

The median age at the time of MBTS surgery was 16 days. Two patients died intraoperatively. Among the 127 neonates transferred to the NICU, shunt failure—defined as either significant stenosis or complete occlusion—was observed in 24 patients, representing an incidence of 18.9%. Demographic and clinical characteristics of the 127 patients according to shunt failure status are presented in Table 3. Shunt failure was significantly associated with lower gestational age (35.6 vs. 38.7 weeks, p=0.001) and lower birth weight (2,406 g vs. 3,061 g, p=0.001). Preoperative and postoperative hematocrit levels were significantly different between groups (p=0.03 and p=0.04, respectively), and postoperative acidosis was more common in the failure group (54.1 vs. 1.9%, p<0.001). Albumin levels were also lower in the failure group (p=0.02). Neonatal mortality was significantly higher among patients with shunt failure (66.7 vs. 11.6%, p=0.001). No differences were observed in creatinine levels or timing of surgery.

**Table 3:** Demographic and clinical characteristics of the study neonates according to shunt failure status.

Variables	Shunt failure (n=24)	No shunt failure (n=103)	p-value
Gestational age (weeks), mean±SD	35.59±2.53	38.68±1.00	0.001
Birth weight (grams), mean±SD	2,406.48±673.35	3,061.58±370.21	0.001
Age on admission (days), median (range)	2 (1–28)	2 (1–35)	0.99
Age at diagnosis (days), median (range)	1 (1–20)	1 (1–35)	0.37
Age at MBTS surgery (days), median (range)	9 (3–300)	16 (1–360)	0.97
Preop Hct (%), mean±SD	41.62±7.43	37.66±6.68	0.03
Postop Hct (%), mean±SD	36.59±5.65	39.50±5.79	0.04
Preop albumin (g/dL), mean±SD	2.80±0.45	3.06±0.38	0.02
Postop albumin (g/dL), mean±SD	2.67±0.34	3.15±0.70	0.02
Preop creatinine (mg/dL), mean±SD	0.64±0.18	0.61±0.37	0.76
Postop creatinine (mg/dL), mean±SD	0.63±0.19	0.60±0.12	0.61
Postop metabolic acidosis, n (%)	13 (54.1)	2 (1.9)	0.001
Mortality, n (%)	16 (66.7)	12 (11.6)	0.001

Data are presented as mean±SD for normally distributed continuous variables and as median (range [min–max]) for non-normally distributed continuous variables; categorical variables are presented as n (%). Continuous variables were compared using Student’s t-test (normally distributed) or Mann-Whitney U test (non-normally distributed). Categorical variables were compared using the chi-square test or Fisher’s exact test, as appropriate. p-values refer to between-group comparisons. Hct: hematocrit; MBTS: modified Blalock-Taussig shunt; SD: standard deviation.

Receiver operating characteristic analysis showed that only preoperative hematocrit had acceptable discriminatory power for predicting shunt failure (AUC 0.767). Postoperative hematocrit, preoperative albumin, and postoperative albumin had poor predictive ability. The optimal preoperative hematocrit cut-off for shunt failure was 36.35%, with 91.7% sensitivity and 51.4% specificity (Youden index 0.431).

In the early postoperative period, pulmonary hemorrhage due to shunt overflow occurred in one patient. Sepsis developed in three patients, and ventilator-associated pneumonia in five. One infant developed acute kidney injury requiring peritoneal dialysis. MODS, NEC, diaphragmatic paralysis, and intracranial hemorrhage with seizures were each observed in one patient.

The overall neonatal mortality rate was 21.7% (n=28). As stated before, two patients died intraoperatively. Shunt-related mortality due to occlusion or thrombosis accounted for 13 deaths (10.0%), while sudden postoperative cardiac arrest was responsible for five deaths (3.9%). An additional six patients (4.6%) died due to sepsis and/or MODS, and two patients (1.5%) succumbed to refractory arrhythmia in the immediate postoperative period. There was no significant mortality difference between patients with univentricular and biventricular physiology. Among infants with syndromic diagnoses, the mortality rate was 5 out of 9 (55.5%).

A total of 101 patients survived to NICU discharge. Long-term follow-up data were available for the majority of these patients. Within the first 24 months after discharge, seven patients died before undergoing a planned second-stage surgical procedure. The causes of death in this subgroup included pneumonia with acute respiratory distress syndrome (n=2), shunt occlusion (n=2), pneumonia accompanied by systemic sepsis (n=2), and one case of sudden unexplained cardiac arrest.

Surgical follow-up records indicated that 57 patients ultimately underwent a bidirectional Glenn procedure. The mean age at the time of surgery was 29.2±23.4 months, with a range from 4 to 70 months. Additionally, five patients underwent primary corrective surgery—including Fallot repair and the Rastelli procedure—at a mean age of 24.4±12.0 months (range: 9–48 months).

In multivariate logistic regression analysis, lower gestational age (OR 0.82, 95%CI 0.72–0.94, p=0.004), preoperative inotropic support (OR 3.46, 95%CI 1.42–8.45, p=0.006), preoperative mechanical ventilation (OR 4.28, 95%CI 1.77–10.37, p=0.001), shunt failure (OR 5.12, 95%CI 1.87–14.03, p=0.001), and postoperative acidosis (OR 6.78, 95%CI 2.19–20.93, p=0.001) were identified as independent predictors of neonatal mortality.

## DISCUSSION

Despite notable advances in neonatal cardiac care, the MBTS remains a cornerstone palliative intervention for neonates with complex cyanotic CHD, particularly in cases in which early definitive repair is not feasible. Contemporary series continue to report substantial early morbidity and mortality after MBTS, particularly in neonates with low birth weight or physiologic instability^3,7,10,11^. In our cohort, MBTS was most frequently employed in neonates with single-ventricle physiology or duct-dependent lesions such as pulmonary atresia with intact ventricular septum (PA/IVS), tricuspid atresia, and variations of double outlet right ventricle (DORV). This is consistent with existing literature, where MBTS is used as a bridge to staged repairs like the Glenn and Fontan procedures^3,11^.

Shunt-related morbidity remained a significant concern in our cohort, with shunt failure observed in 24 neonates (≈19%), comparable to rates reported in prior studies^3^. In our analysis, shunt failure was strongly associated with increased mortality (66.7 vs. 11.6%, p=0.001) and postoperative metabolic acidosis (p=0.001), and it emerged as an independent predictor of adverse outcome. We identified a preoperative hematocrit cut-off of 36.35% as optimal for predicting shunt failure, achieving 91.7% sensitivity and 51.4% specificity with the highest Youden Index. The association between hematocrit and shunt-related thrombosis/occlusion has been described previously, supporting the plausibility of hematocrit-related viscosity and thrombotic propensity contributing to early shunt compromise^12^. This finding is consistent with previous reports that emphasize the prognostic value of hematocrit levels in neonates undergoing systemic-to-pulmonary shunting^12,13^, although exact cut-off thresholds were not specified in those studies. These findings reinforce the importance of early detection, individualized shunt sizing, and optimized anticoagulation protocols in mitigating shunt-related complications. Postoperative antithrombotic strategies vary among centers; UFH followed by antiplatelet therapy remains a commonly adopted approach in neonatal shunt patients^9^.

Several preoperative risk factors—including lower gestational age, low birth weight, and need for mechanical ventilation or inotropic support—were significantly associated with adverse outcomes. These results are in line with prior studies^7,11^. The observed association between higher hematocrit and lower serum albumin with shunt complications may reflect a combination of increased blood viscosity/thrombotic tendency and greater preoperative illness severity in these neonates^12^.

Of particular note, postoperative metabolic acidosis—present in 54.1% of shunt failure cases—emerged as the strongest predictor of mortality in our cohort (OR 6.78, 95%CI 2.19–20.93). This aligns with previous research associating metabolic acidosis with adverse outcomes after systemic-to-pulmonary shunt surgery^7,11^.

While less invasive alternatives like ductal stenting have gained traction, randomized data confirming the superiority of MBTS remain limited. Although ductal stenting is increasingly used, meta-analytic evidence suggests no consistent mortality superiority over MBTS, with potential advantages in selected populations such as shorter hospital stays^8^. MBTS continues to offer reliable pulmonary artery growth and systemic oxygenation for patients ineligible for early corrective surgery.

The overall neonatal mortality rate of 21.7% in our study— comprising intraoperative, shunt-related, sepsis-associated, and arrhythmic deaths—reflects the high acuity and complex needs of our patient population. Interestingly, no significant mortality difference was observed between single-ventricle and biventricular groups, suggesting possible improvements in perioperative support across physiological subtypes.

Syndromic neonates had higher mortality in our cohort, underscoring their vulnerability; however, outcomes may improve with tailored perioperative strategies^14^.

This study has several limitations. First, the retrospective single-center design may limit generalizability. Some potentially relevant variables (e.g., shunt-to-weight ratio or transfusion targets) could not be consistently captured due to the retrospective design. Second, although we performed multivariable logistic regression, the number of mortality events restricts the number of covariates that can be included, and residual confounding remains possible. Finally, postoperative metabolic acidosis and shunt failure may represent early manifestations of the causal pathway toward mortality rather than purely independent preoperative risk markers; therefore, these variables should be interpreted as strong early warning indicators for adverse outcomes rather than solely baseline predictors.

## CONCLUSION

MBTS remains an indispensable palliative option for critically ill neonates with duct-dependent or complex cyanotic CHD. Preoperative clinical and biochemical indicators—such as gestational age, birth weight, ventilatory and inotropic support, hematocrit, and albumin—should inform perioperative planning. Postoperative metabolic acidosis is a strong predictor of adverse outcomes and warrants heightened vigilance. Our findings emphasize the need for individualized management strategies and structured follow-up to optimize outcomes in this vulnerable population.

## Data Availability

The datasets generated and/or analyzed during the current study are available from the corresponding author upon reasonable request.
